# Problems in Psychology

**Published:** 1904-12

**Authors:** Reginald Eager

**Affiliations:** Lecturer on Insanity and Medical Jurisprudence, University College, Bristol


The Bristol

Medico-Chirurgical Journal.

"*Scire est nescire, nisi id me*
*Scire alius sciret.*"

DECEMBER, 1904.

### PROBLEMS IN PSYCHOLOGY.

BY

REGINALD EAGER, M.D. Lond.,

*Lecturer on Insanity and Medical Jurisprudence, University College, Bristol.*

The Presidential Address, delivered on October 12th, 1904, at the Opening of the Thirty-first Session of the Bristol Medico-Chirurgical Society.

THE reason of my being called to occupy this chair to-night has, I am quite sure, been due to an intention on your part that all branches of our learned profession should not be wanting in representation through the person of the President for the year. And so the revolving years have once more brought forward in its turn psychological medicine, which I most unworthily represent, and I may say I feel very deeply the honour you have so graciously conferred on me in nominating me to the presidency of your Society.

It would almost be impossible for me, if not undesirable, to lay before you any thoughts on a particular element of psychology or some abstruse questions connected with it, the time at my disposal being so limited ; but rather, following the example of some of my predecessors, I shall venture to touch lightly upon some few matters which daily experience show me to be real difficulties with my professional brethren, and which prominently assert themselves constantly in a very practical manner, so as to try and see what are the causes of these difficulties, real or ideal.

I am always being assured by the relatives of patients about whom I may be consulted, and very often also by the medical adviser of the family, that the patient is not insane, and cannot on any grounds be considered to be in such a condition ; it may be admitted that he is a little peculiar or eccentric, not more than that, but certainly not insane. Not only to one, who controls a private licensed house and is liable perhaps to be biased, but to those other members of this speciality in charge of licensed hospitals or of asylums maintained for the poor and needy by the counties and boroughs, these patients exhibit almost at a glance in numerous cases the common symptoms of insanity, and a smile tends to creep over our face while we listen to the persistent endeavours of the relatives and friends, and, as I have said, often of the medical adviser, to impress upon one that the case is anything else one likes to call it but insane, as well as at the latter's positive assertion that to sign a certificate would be impossible, as well as morally wrong.

One hears that a gentleman has exhibited for some weeks, or even months, symptoms of low-spiritedness, has apparently lost all affection for his wife and children, makes frequent hints at impending ruin, and one day not long after is found in the office parlour of the firm in which he is managing director and a highly-valued servant, with a loaded revolver in his hand by him on a table at which he is sitting in gloomy despair, saying he has lost everything and must shoot himself. The medical practitioner and the learned consultant called in, and who see him in this condition, agree that he is not insane and that they cannot sign certificates to place him in a place of common safety !

Again, the mother of a lady who is married consults me about her. She informs me that her daughter has gone through a very great deal of trouble caused by the very questionable proceedings of her husband; that this gentleman in an underhanded and incomprehensible way had placed her in an asylum where she was for some two years, and from which the relatives could not get him to remove her. At last, however, the mother on her own motion in some way I did not comprehend, but apparently not with the advice of the physician to the hospital, removed her from the asylum and took her to live with her. She found her perfectly well and quite sane. Still she tells me she has been in a more or less hilarious condition of excitement, not occupying herself at all except a little with music. She is quite controllable by her mother, but she cannot be allowed money, as she gives it all away on unworthy objects or spends it quite foolishly. She will when out walking take off her boots and give them away, as well as articles of her dress also, and return almost bare, she is so good-hearted, charitable and kind. She thought it best to send her into a medical man's house for a time, but being practically without control there she goes out continually to concerts, and listens to bands, and gets excited, and the mother does not think this good for her or that she is so well, and that probably she would be better for supervision in an asylum. She considers there is a very great difficulty in the way so far, as she must impress on me the daughter is in no way insane, only a little eccentric, and that medical men who have seen her declare that on this account she is not certifiable, and ought not to be placed in an asylum.

I forbear to quote more cases, but they are plentiful. These two will suffice to show that we who are trained to this peculiar work see at once that both these cases are unquestionably insane, and indeed that in both these cases there is material enough to found several certificates upon. If this is really the case, how is it that other observers cannot see they are insane as we do? I think little blame can attach to these persons. The features of the disease no doubt are difficult to master, especially if a person has had no asylum experience Still, let us look a little closely into the matter and see if there are other reasons, and some not so excusable. We who are coming in contact with these cases daily can see the insanity clearly and definitely stamped on the patient, in most instances in demeanour, appearance and action, as well as in speech. As Dr. Maudsley has well said, an insane patient utters himself not only by his speech but by his actions. It is very much the same thing as the skilled observer with the microscope, who can with a glance sum up the appearance he sees in the section beneath the lens, whereas the unpractised person will have to puzzle over it for some time to clearly make out its character, and the learner will only see a tangled mass of structure he cannot comprehend, and may take for something it is not, until it is carefully explained to him. There are influences of different kinds which may unwittingly affect and pervert the judgment—no doubt there is a strong feeling that it is prejudicial to the family and to the patient that such "a stigma," as I am sorry to say one still hears it called, as certification should be laid upon the patient, and it is tried, quite conscientiously I am sure, to avoid admitting the patient's lunacy. Some also, I think, are not able to see that what they are willing to look upon as eccentricities—not the true eccentricities of personal fads, manners, or of speech, and tricks of gesture, but the eccentricities which are the remnants of an attack, or the eccentricities which may proceed rapidly to develop into forms of serious mental disease—are a form of disease at all.

Then again there is still amongst us the bugbear as to the responsibility of signing a certificate. I do not mean that proper responsibility which all of us ought to, and I am sure do, feel when we have to perform any special act of our profession, where often indeed the responsibility is far heavier and more serious than in signing a certificate of insanity. I mean the feeling that it is such a risky thing to do, and therefore should be avoided on every ground if possible. Nor am I altogether out of sympathy with this feeling when I remember how in days not long past members of our profession have suffered so unfairly for such an act, even here in Clifton. Still, so far as that goes, the signing of a certificate can often be avoided, we should remember, nowadays, by placing the patient in the voluntary boarder class, and so obtaining fitting treatment.

I think it will be admitted that any grave deviation from the normal in an organ of the body, even though temporary, is disease; so also must it be with the brain. We must also admit that any palpable deviation from its normal condition as exhibited by the emotions, the intellect, and the will, is insanity, though the state exhibited may not imply the necessity of treatment under restraint. It must surely be admitted by an unbiased person that when inflammatory changes in the brain are taking place, producing such results as I have typified, and leading, as is always the case, to more or less disturbance of the functions of sleep, respiration, digestion, and changes in other organs, it becomes a very serious question as to what action a medical man should take, and whether he is not bound in such a case to give every aid in the way of specially skilled nursing, treatment and appliances, such as those well versed in the treatment of the disease do not hesitate to say can only be found in a fully-equipped asylum. Has it not struck you at all as a terribly sad thing—to me it is one of the most awful and sickening horrors of the newspapers—to see carefully recorded day by day the tale of suicides, homicides, and murders, in most of which we find recorded that depression or similar symptoms had lately been observed; a very large number of which unquestionably were avoidable had medical men only acted with the truest kindness in getting these poor creatures placed under asylum treatment previously. I know well the many difficulties there often are in the way, but my experience leads me to feel that so much more might be done. I also know that if a medical man is only firm in his expression of his opinion as to the danger involved in all such cases of depression, that opinion is rarely put on one side and not acted on, except perhaps in the case of members of the upper classes of society, who often think they know better than their medical adviser. In such cases one pities the folly of their conceit.

Sometimes it is felt that the patient is unquestionably insane, but the evidence upon which to found a certificate seems hazy and insufficient. When the certificate is written it will then probably be found that sufficient "facts" have been stated to form the foundation of several certificates. It is not sufficiently understood, I think, how very little is really needed to form a good and valid certificate. The bare statement that the patient is evidently greatly depressed, religiously or otherwise, and has attempted or is actually contemplating suicide, is as strong a certificate as a man can write, and far stronger than many certificates in which all four lines and the margins of the document beside are filled up with an involved statement of "facts."

But there is another cause to which I have referred which often prevents certification. It is the fear of the law courts. This fear, however, I hope and trust is a thing of the past and has no basis nowadays. No doubt it takes a long time to eradicate such a dread, a dread which before 1890 was one which indeed had grown to be a matter of a very serious nature. Since the passing of the Consolidated Lunacy Act, called that of 1890, that dread has been set at rest, and medical men can so far do their duty fearlessly. From observations made to me from time to time, I am very sorry to say that medical men do not seem to find time to keep up their acquaintance with that part of medical jurisprudence which treats of the Lunacy Act. Probably very few medical men own a copy of the Act or think of looking at it in *The Medical Directory;* nor, I fear, do they even look at the printed advice and regulations which the Act now attaches to the present form of certificates. Anyhow, the Act has provided a simple but sufficient, and I am almost inclined to say perfect, safeguard. Since the Act came into force the attempts to prosecute may be counted on the fingers of one hand, and each case has hopelessly broken down. So much is this the case, that I understand no lawyer nowadays would dream of bringing an action against a medical man for signing a certificate.

The reason for this and the protection so amply afforded by the Act my experience shows me is but imperfectly understood. In the first place there is the declaration by the Act as to bona fides on the part of the medical man signing—barring prosecution. In the next place I wish to call to your minds that when the petition and statement are prepared, and the two medical men have properly filled in and signed their documents, these are all taken to a magistrate duly appointed under the Act for the purpose. He sees these documents and examines them. If he likes he can see the medical men, and he can see the patient or not, as he pleases, before he signs the "order" for admittance into an asylum. Some justices have an objection to seeing the patient, and some, on the contrary, make a point of always seeing the patient. In some very exceptional cases it may be desirable, no doubt, that the patient should not be visited and perhaps dangerously excited by the visit of the magistrate. However, in my opinion the medical attendant or medical man signing certificates should make a point of getting the magistrate to see the patient, for it is in his seeing the patient that the great safeguard lies, although the magistrate's act is simply judicial. If a magistrate appointed for the purpose examines the two certificates, sees the patient, finds the patient's symptoms tally with the medical men's statements, and then signs the order, it renders it next to impossible with that backing for any one to urge that the two medical men acted without proper care and bona fides, and no action could lie.

The medical man and the friends of a patient may have an idea that residence in an asylum is a thing so dreadful that it must be avoided at all costs, even to the length of breaking an Act of Parliament, as has been urged, and the lunatic is sent to the care of totally irresponsible and unauthorised persons in numerous cases. A great deal of late has been heard of "borderland insanity" in order to get over the difficulty. What are borderland patients? As I understand, they are patients whom one must not call insane. But if so, where are we to draw the line between sane and insane? They are neither fish, flesh, fowl, or good red herring. They are not cases, it is said, fitted for asylum treatment, and asylum treatment is entirely deleterious and positively harmful to them, and will excite or depress them until they actually become insane. If there is the least truth in such a theory, it is a very terrible matter indeed, because then surely the truly insane also cannot benefit, as we can show they have done for years past, by asylum treatment. These patients must react for ill on each other, and the sooner every asylum in the kingdom is pulled down the better. Remembering the kindly-hearted and humane men who have managed these houses for years past, and done so much in every way for the improved treatment of the insane—such men as Dr. Conolly, of Hanwell, and the first Dr. Fox, at Brislington—such men would surely have tried to pull down the one and close the other if there had been any truth in the statement. It is a well-known fact that no lunatic has ever been known to have been the worse by contact with others. Again, when does a borderland case cease to be one and cross the border? I know indeed full well that insanity is developing in numerous cases before ever it is possible to raise a question of legally sane or insane, and long before the friends are the least aware of it or the medical man consulted. This incipient insanity I understand just as I do the incipient stages of other diseases. Is that a borderland case when a man recovering from a fever shows symptoms of depression only for a few hours, with no evident delusion, and yet is dead in a few days with his throat cut? Does it mean the elegant young lady, morally weak and ill developed, who gets away from her friends on an opportunity, roams about London and plays Messalina for a week? The phrase has been applied to these cases, and it is urged that the right treatment is that they should be supervised in a special department of an ordinary hospital—this necessarily as a free agent, for if confined or restrained the question of the liberty of the subject will arise; or in charge of some benevolent gentleman and his wife with small means, not at all necessarily having medical knowledge or a house adapted for such treatment, to see if the case develops into insanity. Can there be a borderland condition to a patient inoculated with the germs of scarlet fever or small-pox? Can a case with harmless delusions be on the borderland of insanity? Does it only become a case of certifiable insanity when the patient's delusions lead to their giving away their valuables to unworthy recipients or to others than their immediate relatives? There are also persons whose brains have never been quite fully developed, but who perhaps get on fairly well up to the age of puberty, when they break down. They are often sources of annoyance and vexation on many grounds to their relatives in the way of their talk, behaviour and general conduct. However treated, little good comes of it, but rather outbreaks of viciousness from time to time. Are such to rank as borderland cases? Some say so. It is very difficult to express in words what one feels with regard to this borderland theory, and one needs to place restraint on one's self to refrain from saying what one feels is the basis of the whole thing. To say the least of it, in numerous cases, we will hope unwittingly, it is nothing less than a cruel deception; and so the patient who has passed even the incipient stage of an attack, and who needs at once everything that experience has shown is necessary to cut it short and lead it into paths of recovery, is practically deprived of this chance by being placed in a position where there may be no medical supervision, in a house carefully secluded from observation of others, and where there is a want probably of all proper attendance and staff. I am unwilling to trouble you with examples, no doubt some occur to you, and the following will suffice. A young lad was considered to be suffering from borderland insanity. To the trained observer he was really an imbecile. The lad's mother and family were glad to have such a soothing definition of his malady given them, as well as at being told an asylum would be the worst place possible in which to place him. They placed him with a man and his wife, persons of small means, with no medical knowledge or even skill in nursing, in an out-of-the-way house in the north of London, where they were led to suppose he would soon be well. He was there some years. When visited he was often found left locked into the house alone, with no one near, there being no house within half a mile. No doubt severe remarks were made at this condition of things by the parents, still on one occasion he set fire to himself and got burnt, and on another he fell down off a table on to which he had got to look out of the window and broke his leg, and I found him in a garret for his bedroom, given him, it was said, because it was so airy and he could see all round the country-side from the window. He was removed to asylum treatment, but too late: he had drifted into a state with little hope of amelioration. Had this treatment been given at first much might have been done to improve his condition.

There appears to be a happy tendency in the present day amongst all classes of society in serious forms of disease to enter a hospital for treatment and nursing, and with the rich the private hospital and nursing home are most acceptable. And why is this? I suppose it is because it is becoming generally understood and accepted on all sides that even in the dwellings of the rich, with all that money can provide, an ordinary house is not equipped in a way that is suitable to the treatment of such cases. The hired nurses, however good, are often not the same as when under hospital control and direction, and a skilful authority is at hand in the smallest emergency. Many of the conditions of a private house are most undesirable at such a time, and there is a great want of that battery of drugs and appliances always at hand in a properly constituted hospital, however small. If we look at matters in this way in ordinary medicine and surgery, and even create hospitals for treating special forms of disease, why is the poor lunatic to be the only one who is to be deprived of all the aids which years of devotion to the work, of thought and consideration, have built up into the special hospital for his form of disease; where the physicians have been laboriously drilled and specially qualified for their anxious work by a very trying and abstruse training; where the nurses are also specially trained into habits of unremitting patience and endurance amounting to an exhibition of a virtue, and where everything is adapted to security from harm, as well as from restraint; and where the only and one object is the effecting a cure, and the whole tone of the house is such as will bring to bear upon the patient, probably quite imperceptibly to himself, every circumstance most effective to that end? When a man unluckily develops a tænia and gets a cysticercus in his brain, producing unpleasant symptoms and paralysis, everyone will agree that the best thing for him to do is to go to a hospital, public or private, and he will be pressed so to do. When the cysticercus in his brain produces symptoms of mania then the endeavour of everyone is to prevent his being treated in the place most appropriate for his case. Treat him as a borderland case—yes, certainly, and send him to Mr. So-and-So, who receives "cases" into his private house. Surely the true treatment should be to send him to a hospital also, but it should be a lunatic hospital, public or private. A broken leg as a matter of course is taken to a hospital, and to be logical surely a broken brain requires a similar and appropriate treatment. Cases sickening for fevers or other serious complaints are very sick people, and require anxious and trained nursing; but I know no such anxiously sick people as those who are showing early symptoms of mental attack, or who more certainly require every aid applied as soon as possible to the relief of the morbid brain condition; and no better aid can be obtained than the special hospital treatment of an asylum, with all its circumstances of curative rest and calm, and its expert and systematic treatment. No class of cases require the attention of the trained nurse more than the subjects of incipient and recent insanity. The constant observation by such trained persons is invaluable amongst the insane, many of whom it must be remembered can give no indication of symptoms of disease of the general system, and where clinical phenomena so often require to be interpreted much in the same way as those of a sick child or even of a sick animal.

Such a happy condition of treatment those who look at the matter from my standpoint do not think it is possible for them to obtain as single patients dotted all over the country at random, whether as single patients under the Act or kept unquestionably in numerous cases surreptitiously in evasion of the Act. It is not that in many such cases their bodily comfort is not well looked after, for in some cases it is luxuriously so, and they receive every good and kind attention. The family are probably considerate and good-natured to them, and give them a good deal of companionship. The ordinary and general occupations and amusements of the family the patient shares in as far as possible, and if not so far capable, often—indeed, far too often—is allowed to drift into his own methods. Now no one can say this is treatment, much less treatment of a kind likely to prevent so-called borderland cases drifting into that which this treatment is supposed specially to avoid, a true attack of insanity. But such very sick, impotent folk cannot be treated with justice thus. They need in their way the full appliances of an institution, and the freedom from irritation of relatives and friends, just as much as a case of stone in the bladder or small-pox. Why then are not the insane to be treated in their own hospital?

At present, and I fear for some time to come, the treatment of insanity remains under the direction and rule of an Act of Parliament. The sins of the fathers are being visited on the children to the third and fourth generation. For had not our public institutions in the earliest years of the past century been such sinks of abomination and mismanagement such as the Royal Commission of that period showed them to be—and let it be remembered the private licensed houses were not included in this condemnation—I suppose we should never have had the many Lunacy Acts that were passed. It has always appeared to me to be a very stupid and clumsy arrangement that certificates of insanity should have to be signed and used at all. I cannot see why any person suffering from incipient and fully-developed insanity cannot be taken into a hospital for treatment just as easily as the man who has fallen from a ladder in the street and fractured his skull. He no doubt might if he were able, or liked, to protest against such treatment, yet willy-nilly he is taken. There is only, of course, one requirement that should be obligatory, and that is that the hospital must be one fully equipped for the treatment of such cases. Nor do I think that the huge palaces—for such they are, palaces such as few very rich men even could build, holding one to two thousand insane persons, largely incurable—are desirable places for treatment of the curable insane.

The time will come, I hope and trust, when we may see each town of a fair size having an ample and large plot of park-like ground of many acres, well wooded, with lovely views, and laid out so as to extend and amplify its beauties, whilst here and there parterres of flowery plots and choice foliaged plants may add to its attraction. There would be many tame animals about and birds in spacious aviaries. There would be a central institution, roomy and decorated quietly, but still with ample luxury and decorative art. In this house would be protected, taken care of, and treated, the suicidal, homicidal, and acutely excited cases. It would not be very large in its dimensions, but possess an ample staff of efficient nurses. There would also be small cottages, villas, and larger dwelling-places fully adapted to their purpose in different parts of this park for the quiet and harmless cases. Work and employment of all kinds suited to the patients' needs could be provided for such as were entering on the earlier phases of amendment and recovery, and who now could begin to make use of them. No greater mistake has been made than to suppose the insane can employ themselves before then. When a patient begins to occupy himself in some way then you may be sure he is entering on the stages of amendment, if not of cure. The so-styled amusements provided would all be of the soothing and quiet kind, not frequent or exciting, which is too often the case with music and dancing. In my opinion also a great mistake is made in this matter. It is an error to place an excited crowd in conditions of excitement. The imbeciles and epileptics would be in separate parts of this domain and grouped in separate houses. Hysteria, chorea, intricate cases of nervous disease, &c., could well be treated here also by their own medical attendant. One point there is that must be provided for under such circumstances. In compelling a patient to take food necessary to preserve his life, to dress and wash, and when indecent and violent behaviour of any kind leads to make restraint or seclusion necessary, and when the tendency to suicide and homicide requires the constant presence of a restraining nurse, and in such cases as have to be dealt with by forcible means and interference with their personal wishes, we arrive at that very ugly condition of which so much is made by an excited British public—that considers itself fully qualified to sit and judge upon any subject, however learned or intricate—"interference with the liberty of the subject," the common view being that such a thing cannot be allowed on any account, though the British matron is quite ready to shut up her children even of grown-up age in their rooms without food for some hours as punishment as well as to beat them black and blue, and the master to cane a youth at school. Yet when it becomes a question of whether a man is to cut your throat or blow his own brains out, then if means are taken to prevent him one is liable to an action at law and *habeas corpus* and all that kind of thing if it is done before a certificate of insanity is signed.

How then could such cases be dealt with in such circumstances. Every patient that arrived at the establishment would be seen at once by the physicians attached, and any cases found to be in these conditions would be detained until they had been seen by a justice of the peace, who would sign an order for their treatment in that part of the place which I mentioned as being built and adapted especially for such cases, and similar action would be taken with any case falling into such a condition whilst under treatment in the other parts. This would do away with certificates and all the vexing and trying methods now in use, and make prompt treatment as easy for insanity as for any other disease. In the general hospital the patients are under a certain rule as to going out of the hospital, receiving friends, &c., and so would insane patients be. None would be dealt with by the magistrate, and the central hospital, except those whose condition renders it impossible to treat them otherwise with due care for their safety, and in this way the liberty of the subject would be fully conserved and guarded.

From the days of Sir Thomas More until now we have all liked to write up our Utopias. Some Utopias have, however, come into existence, and the Utopia I have sketched will, I think, probably come into being in the not so far distant future.

